# Will pay-per-view in the advanced charging model recover the business losses for online video companies in China?

**DOI:** 10.1371/journal.pone.0297977

**Published:** 2024-01-31

**Authors:** Congmin Zhang, Seuk Wai Phoong

**Affiliations:** 1 Institute for Advanced Studies, Universiti Malaya, Kuala Lumpur, Malaysia; 2 Department of Decision Science, Faculty of Business and Economics, Universiti Malaya, Kuala Lumpur, Malaysia; University of Naples Federico II: Universita degli Studi di Napoli Federico II, ITALY

## Abstract

The evolution of Internet technology is closely mirrored by the innovative business and profit models emerging within the platform economy. Integrating this economic framework, online video platforms are dynamically refining their pricing strategies to optimize profit margins. This paper, anchored in the theoretical construct of second-degree price discrimination, selects the Chinese online video platforms iQiyi and Tencent Video for in-depth case studies. It charts the progression of its pricing strategies and juxtaposes these with those of prominent international online video sites to highlight both congruities and divergences. The synthesis of theoretical models with real-world case studies culminates in strategic recommendations to foster the growth of Chinese online video platforms in the global Internet arena.

## Introduction

Internet companies leverage technological advancements to consolidate resources, creating platforms that transform traditional economic models and give rise to Internet platform financial enterprises [1]. This shift marks a departure from the Industrial Revolution, dominated by the industrial manufacturing industry. The platform economy wields more transformative power, ushering in significant political, social, and business changes [2]. In the face of a dynamic market with novel demands, opportunities, and a high attrition rate within the network economy, platform enterprises must adopt new digital strategic thinking. They need to develop digital competencies and devise business models that are both dynamic and optimized, capable of rapid adaptation to maintain market competitiveness [3].

The business model, a concept of value creation, requires companies to identify their niche and generate revenue by surpassing technological limitations. They must engage in dynamic competition with their value chain partners and deeply understand customer expectations and preferences. Companies deliver goods and services to customers through the value chain, aiming to maximize profits [4]. In the context of the digital economy, the Internet of Things, platform companies and digital services are integrated [5], and digital platforms innovate value creation and value acquisition models through market reorganization [6].

Evans delineates three categories of platform companies across various industries [7]. The first, market-makers, facilitate transactions between multiple members, with e-commerce platforms like eBay and Taobao as exemplars. The second, audience makers, match advertisers with audiences, capitalizing on customer accumulation to attract advertisers, as seen in traditional print media, free television, and portal sites. The third, demand coordinators, create network effects among two or more groups through goods or services, exemplified by Apple’s software marketplace and electronic payment systems.

According to the "The Biggest Companies in the World in 2021" [8] report by Pricewaterhouse Coopers (PwC), seven out of the top ten companies by market value belong to the platform economy, including PC operating platforms like APPLE INC’s iOS and MICROSOFT CORP’s Windows; mobile operating systems and application stores like ALPHABET INC’s Android system and Google application market, APPLE INC’s iOS mobile terminal, and Apple Store; and Internet application platforms such as AMAZON, FACEBOOK, TENCENT, and ALIBABA.

At the end of 2019, the novel coronavirus outbreak triggered a global economic recession. Despite this, the platform economy exhibited remarkable resilience. As a dynamic force within this economy, China showcased rapid development and a concentration in specific fields that merit further investigation [9]. The 49^th^ "Statistical Report on Internet Development in China" [10], notes that by December 2021, the number of Internet users in China had reached 1.032 billion, with 975 million engaging with online video services (including short videos), representing 94.5% of total internet users. Continuous innovation in business and technology has spurred the growth of China’s online video market.

iResearch has defined the online video industry as a sector where videos are uploaded by users, licensed from creators, or produced by companies and are available for free or paid viewing and downloading on internet platforms [11]. This research focuses on comprehensive online video companies in China that predominantly use "PUGC—Professional + User Generated Content" and "OGC—Occupationally-generated Content" for content creation, excluding those solely reliant on "UGC—User Generated Content." The content primarily consists of short and live videos. In 2021, China’s online video market revenue was approximately 131 billion yuan, with significant contributions from Baidu, Alibaba, and Tencent’s platforms: iQiyi, Youku Tudou, and Tencent Video. Data from Statistics as of January 2022 indicates that the leading online video platforms in China by monthly unique visitors are iQiyi (168.29 million), Tencent Video (157.71 million), and Youku Tudou (139.02 million) [12].

Despite a substantial user base, online video companies have consistently incurred losses. Alibaba’s financial statements reveal that between March 31, 2019, and March 31, 2021, the digital media and entertainment segment’s adjusted EBITA were -16.226 billion yuan, -11.446 billion yuan, and -6.118 billion yuan, respectively. iQiyi’s official financial data from 2016 to 2021 reported revenues of 11.24 billion yuan, 17.38 billion yuan, 24.99 billion yuan, 28.99 billion yuan, 29.71 billion yuan, and 30.55 billion yuan, operating costs of 11.44 billion yuan, 17.39 billion yuan, 27.13 billion yuan, 30.35 billion yuan, 27.88 billion yuan, and 27.88 billion yuan, and net profit of -3.074 billion yuan, -3.737 billion yuan, -9.061 billion yuan, -10.28 billion yuan, -7.007 billion yuan, and -6.109 billion yuan, respectively [13]. The net profits were significantly negative, highlighting the need for a strategic shift. iQiyi CEO Gong Yu has indicated a strategy overhaul, prioritizing profitability over market share, reducing costs, enhancing efficiency, and discontinuing unprofitable ventures, discontinuing its past strategy of cornering market share via various products, which did not result in net positive benefits.

"2021 China Internet Advertising Insights" by Quest Mobile observed growth in China’s advertising market from 2019 to 2023 (forecasted), yet revenues from advertising for internet video companies are declining [14]. Consumer aversion to ads is a well-documented phenomenon, with the perceived value and cost of ad viewing affecting their willingness to engage with the main content, per Kim et al. [15]. The entertainment value, informational content, and annoyance factor of ads can influence viewer attitudes, as posited by Yang et al. [16]. Shon proposed strategies for boosting market share among online video companies include reducing ad length and adapting to consumer preferences [17]. Wang et al. suggest that the challenge for these companies lies in converting free users to paying subscribers, given that online video advertising comprises only a minor fraction (4–5%) of the overall Internet advertising market, trailing significantly behind e-commerce advertising (~39.6–48%) [18]. From the growth rate perspective, the number of ads placed on online videos is much lower than in short videos. In terms of increasing content revenue, the content supply of video platforms is unstable and insufficient due to the pandemic’s impact. The increase in content revenue has also been hampered by the value of cash transactions and barter transactions, such as the high cost of copyright acquisitions contradicting cost reduction strategies.

In order to increase the company’s profits, the company adopts a price discrimination strategy and adopts differentiated pricing according to the different price expectations of different customers [19]. When companies obtain consumer preferences, they can adopt price discrimination pricing [20]. Currently, the strategies to create profits for Chinese Internet online video companies and finding suitable profit models and pricing strategies are still urgent issues that need to be solved.

Data on China’s online video sites [21] for 2022 identified the most frequented platforms as iQiyi (168.29 million), Tencent (157.71 million), Youku (139.02 million), and Sohu (127.96 million), with iQiyi and Tencent Video leading in popularity. Notably, Youku Tudou’s viewership data remains undisclosed by the Alibaba Group. iQiyi and Tencent Video have simpler pricing models and fewer bundled fees than their parent companies’ other business segments. Consequently, this research has selected iQiyi and Tencent Video for case studies. It aims to develop a profit-maximizing pricing model using secondary price discrimination within a two-part pricing strategy. The goal is to refine the profit models for business model transformation in Chinese internet video companies, offer insights for devising viable pricing strategies, and expand the theoretical framework of pricing strategy.

## Literature review

The burgeoning growth of platform economy enterprises is primarily attributed to advancements in Internet and network communication technologies [22]. Evans contends that the platform economy is instrumental in forging an efficient market, emblematic of innovation in business models during the digital age, and regards it as a distinctive expression of a bilateral market within the framework of traditional market economics [23]. Through the integration of communication, energy, and transport facilitated by the Internet of Things (IoT), this economy is on the brink of achieving near-zero marginal costs [24]. Additionally, various providers offer differentiated services, bridging gaps in information resources and fostering expedited development [25].

Scholarly research on the platform economy has identified two pivotal characteristics. The first is network externalities, which increase the propensity to purchase as the number of similar units sold grows [26]. Armstrong elucidated that the network effects within the platform economy mean that the value derived by users on one side is affected by the user base on the opposite side—the larger the user base, the greater the derived utility. The second characteristic is the concept of a two-sided market [27]. Rochet and Tirole’s examination of platform firms within such a market revealed that platforms can profit from transactions between numerous users [28]. In 2004, Rochet and Tirole suggested that platform enterprises devise pricing structures that incentivize participation from both customer segments by charging higher fees to one segment while minimizing costs for another, thereby influencing transaction volumes and the market’s two-sided nature [29].

The digital economy has forced companies to forge business models by combining the digital environment, the applications of new subscription-based pricing and sales models changed the way of generating profits [30]. By purchasing copyright merchant video platforms, the main sources of income for companies are advertising, membership fees and some paid videos [31]. Artero compared the profit models of YouTube and Hulu, the well-known online video sites in the United States (US) [32]. Video-on-demand is an acceptable service among customers, which will be used broadly [33]. Both companies started by providing content to users for free and monetized internet traffic by attracting more users, which increased the viewing volume of audiovisual content. Hu analyzed and compared typical video websites in China and other countries and concluded that the main profit models are advertising, membership services, and video malls [34]. Kweons’ comparative study of main online video websites in the world like Netflix, Amazon Prime, Disney+ and Hulu Plus predominantly relies on advertising, pay-per-view, and subscription models as their principal revenue streams [35].

## Methodology and model

### Price discrimination

In neoclassical economics, manufacturers employ diverse pricing strategies to attract more consumers in various market types. Pigou introduced the theory of price discrimination, which involves a seller charging different prices to different consumers for the same goods or varying prices to the same type of consumers based on the quantity or frequency of their purchases [36]. This approach to pricing takes several forms: first-degree, second-degree, and third-degree price discrimination. First-degree price discrimination maximizes firm profits by setting individual prices for each consumer, effectively capturing the consumer surplus as profit [37, 38]. Third-degree price discrimination involves segmenting the market based on customer characteristics and adjusting commodity prices accordingly [39]. Meanwhile, second-degree price discrimination reduces the unit price of goods as the quantity purchased increases, such as in a two-part pricing system where enterprises set a marginal price equal to the marginal cost and generate profits through a fixed fee [40].

Lewis clarified that the two-part pricing system’s core is the consumer’s requirement to pay a fee directly proportional to their consumption plus a portion covering indirect expenses [41]. Oi referred to Disney’s approach [42] as an example, where a two-part pricing system that applies differential treatment maximizes profit from an individual customer. This model sets the pay-per-view price at a marginal cost, and different customers are charged varying admission fees. Oi also examined optimizing a two-part pricing system to maximize profits by charging customers uniform entrance and per-use fees, thereby avoiding discriminatory pricing.

There are a few assumptions in the Oi model: *Q* is the number of visitors, *T* is the visitor admission fee, *P* is the pay-per-view product price, *C* is Disney’s cost, *MC* is Disney’s marginal cost, *MR* is Disney’s marginal revenue, *R* is Disney’s total revenue, and *MC* is a given value.

**Fig 1:**
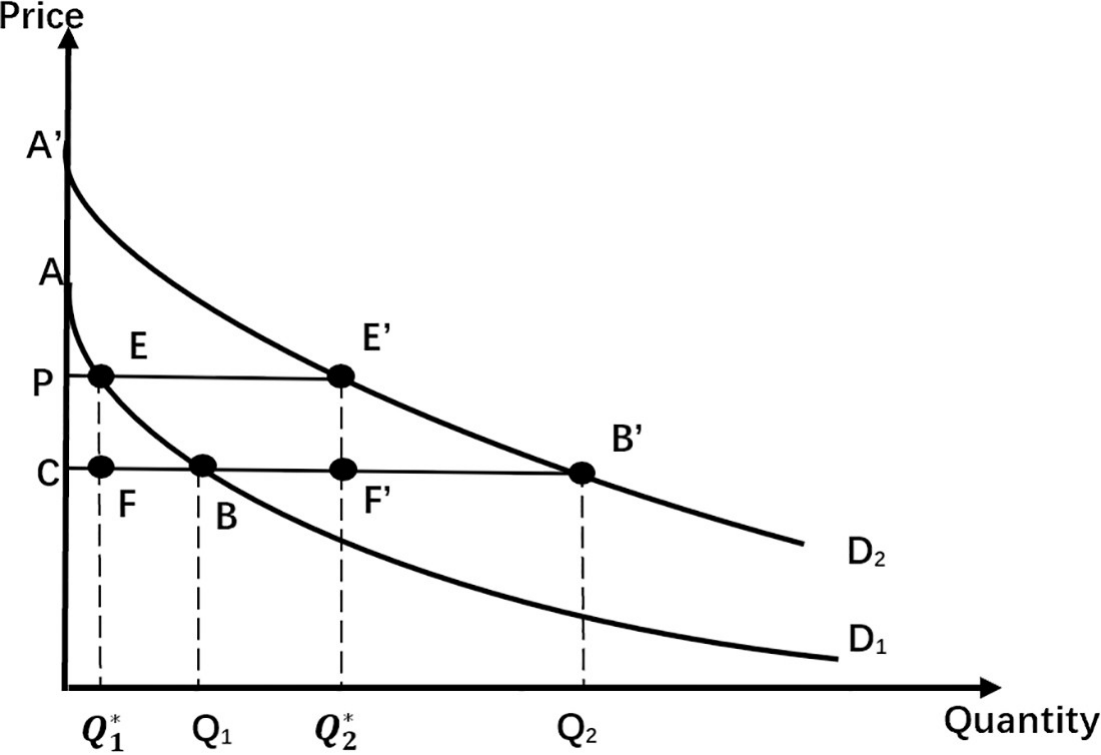
Two types of consumers with constant utility demand curve.

Case 1: Two Types of Consumers with Constant Utility Demand Curve (Fig 1)

Assuming that the consumer’s income elasticity is zero, the effect of the demand curve on income and the one-time entry fee is constant (consumer surplus equals the area below the demand curve and above the price line). The demand curves of the two types of consumers are D_1_ and D_2_, which are constantly utilized and have zero income elasticity. D_1_ represents low-value customers, which means that when Disney sets a fixed entrance fee, D_1_ has lower demands than D_2_. However, D_2_ is for high-value customers, and the customers on this curve are willing to pay more for entrance fees than D_1_ when they are in the same quantity. This case assumes that the Entrance Fee, *T*, does not exceed the smaller amount of the surplus of the two types of consumers. Considering one kind of consumer surplus is the area of *ABC*, and the other type of consumer surplus is the area of *A*′*B*′*C*, then the pay-per-view price, *P*, is equal to the marginal cost *MC*, at which point no profit will be generated.

Assume that all of Disney’s profits come from ticket fees and:

$$\pi =\pi_{1}+\pi_{2}=2ABC$$ (1)

Changes in profits from the first type of consumers:

$$\Delta \pi_{1}=\pi_{1}^{*}-\pi_{1}=AEP+PEFC-ABC=-EBF$$ (2)

Changes in profits from the second type of consumers:

$$\Delta \pi_{2}=\pi_{2}^{*}-\pi_{2}=AEP+PE\prime F\prime C-ABC=EE\prime F\prime B$$ (3)

At this time (EE′F′B) > (EBF), Disney’s profit changes as:

$$\Delta \pi =\Delta \pi_{1}+\Delta \pi_{2}>0$$ (4)

**Fig 2:**
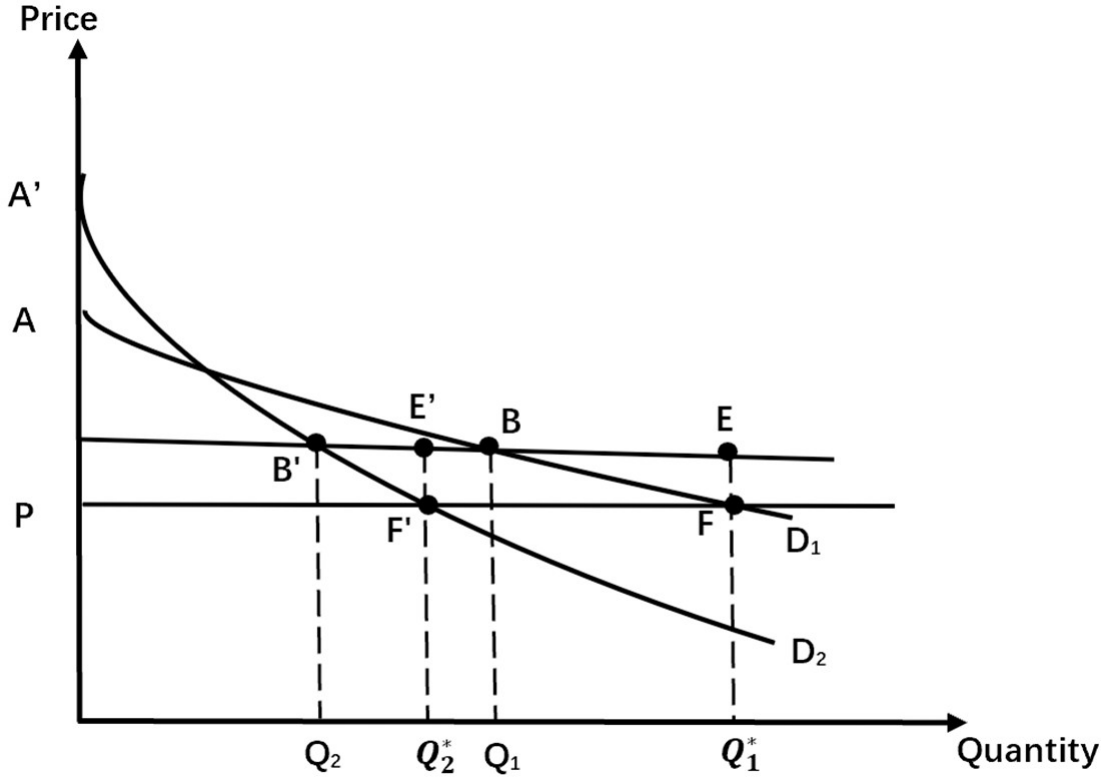
Two types of consumers with the demand curve overlapping.

Case 2: Two types of consumers with the demand curve overlaps (Fig 2)

In this case, concocting utility functions with the usual convexity properties, the consumer demand curves overlap, and the income effect remains zero. When the monopolist sets the pay-per-view price, *P*, equal to the marginal cost, *MC*, and raises the Entrance Fee, *T* (with the condition that the fees cannot be more than the value of the lesser surplus of the two types of consumers), the profits are all attributable to the entrance fee income of the two types of consumers:

π=ABC*2 (5)

If the pay-per-view price, *P*, is set lower than the marginal cost, *MC*, the price will result in a loss in the pay-per-view fee of the entertainment facility, but the increased Entrance Fee, *T*, will make up for the part of the loss.

Changes in profits from the first type of consumers:

$$\Delta \pi_{1}=\pi_{1}^{*}-\pi_{1}=AFP-CEDP-ABC=-BEF$$ (6)

Changes in profits from the second type of consumers:

$$\Delta \pi_{2}=\pi_{2}^{*}-\pi_{2}=AFP-CE\prime F\prime P-ABC=E\prime BFF\prime$$ (7)

If (*BEF*) < (*E*′*BFF*′), the monopoly remains profitable.

### The optimal two-part pricing system

Tang and Li deduced the optimal two-part pricing formula from the two-part pricing model [43]. Assumptions are as follows:

There are two types of consumers, 1 and 2, in the market. They have different preferences *x* for the same product; the higher the *x* value, the greater the desire to buy. *σ* represents the preference of type 1 consumers, while 1 − *σ* represents the preference of type 2 consumers. Consumers of the same type have the same linear demand curve—the marginal cost of a monopoly producer *MC* = *K*, where *K* represents a given constant.

Consumer surplus is expressed as:

$$U=xV_{\left(n\right)}-A$$ (8)

Where *V*_(*n*)_ represents the utility obtained by purchasing the commodity, which is expected to be the same for all consumers, and *U* represents the price paid for the purchase of goods. Under the two-part pricing system, *A* = *T* + *p*_*n*_, *T* means the entrance fee, *p*, is the pay-per-view price, which is the marginal price, and *n* represents the quantity demanded.

Per the assumption of *V*(*n*), construct a quadratic function:

$$V_{\left(n\right)}=-an^{2}+bn$$ (9)

$$V_{\left(n\right)}\prime =-2an+b$$ (10)

If consumers decide whether to consume for the first time depending on the entrance fee, *T*, the pay-per-view price, *p*, will not affect the entry demand. Moreover, once consumers pay the entrance fee, the need for pay-per-view is a peripheral variable and is not considered. Deriving first-order conditions:

$$xV_{\left(n\right)}\prime =p$$ (11)

Taking *V*_(*n*)_′ = −2*an* + *b* into (11), the personal needs of each type of consumer becomes:

$$D_{i}\left(p\right)=\frac{b}{2a}-\frac{p}{2ax}\left(i=1,2\right)$$ (12)

The total demand for the product is:

$$\begin{array}{ll} D_{i}\left(p\right)\prime & =\sigma D_{1}\left(p\right)+\left(1-\sigma \right)D_{2}\left(p\right)\\ & =\sigma \left(\frac{b}{2a}-\frac{p}{2ax_{1}}\right)+\left(1-\sigma \right)\left(\frac{b}{2a}-\frac{p}{2ax_{2}}\right)\\ & =\frac{b}{2a}-\left(\frac{\sigma }{x_{1}}+\frac{\left(1-\sigma \right)}{x_{2}}\right)\frac{p}{2a} \end{array}$$ (13)

Letting $\frac{\sigma }{x_{1}}+\frac{1-\sigma }{x_{2}}=\frac{1}{x}$, then:

$$D_{\left(P\right)}=\frac{b}{2a}-\frac{p}{2ax}$$ (14)

The net consumer surplus is:

$$\begin{array}{ll} NU_{\left(p\right)} & =x_{i}V\left[D_{i}\left(p\right)\right]-pDi\left(p\right)\\ & =\frac{{\left(x_{i}b-p\right)}^{2}}{4x_{i}a} \end{array}$$ (15)

Consumers decide to purchase pay-per-view items regardless of the fixed entrance fee. Assuming that the second type of consumer pays more, there is more consumer surplus. Monopoly firms set prices such that entry fee ≤ low-paying consumer surplus. Then:

$$T\leq NU_{\left(p\right)}=\frac{{\left(x_{1}b-p\right)}^{2}}{4x_{1}a}$$ (16)

Assuming that the firm’s fixed production cost is *C*, the profit function can be obtained:

$$\begin{array}{ll} \pi & =T+\left(p-K\right)D_{\left(p\right)}-C\\ & =\frac{{\left(x_{1}b-p\right)}^{2}}{4x_{1}a}+\left(p-K\right)\left(\frac{b}{2a}-\frac{p}{2ax}\right)-C \end{array}$$ (17)

The first derivative obtains the optimal marginal price at the time of profit maximization:

$$p\prime =\frac{C}{2-\frac{1}{x\left(1-\frac{x_{1}}{x_{2}}\right)-\frac{x_{1}}{x_{2}}}}$$ (18)

Taking *p*′ into the entrance fee, the optimal admission fee can be obtained from:

$$T\prime =\frac{\left\{x_{1}b-\frac{C}{2-\frac{1}{\sigma \left(1-\frac{x_{1}}{x_{2}}\right)+\frac{x_{1}}{x_{2}}}}\right\}}{4x_{1}a}$$ (19)

In the model discussed, it is apparent that the entrance fee pricing primarily hinges on the demand function of consumers with a lower willingness to pay. On the other hand, the optimal price for commodities is determined by marginal costs, the distribution of consumers across different payment thresholds, and consumer preferences. Suppose consumers sensitive to lower costs represent a significant segment. In that case, monopolistic vendors typically opt for a pricing model with a lower entrance fee to entice a broader base of consumers to pay a fixed charge. Conversely, a strategy of low entrance fees coupled with higher pay-per-use charges may be employed to capture a greater consumer surplus from those willing to pay more.

Long posited that the two-part pricing system is especially pertinent to monopolistic industries. This system allows monopoly firms to seize a larger share of consumer surplus and maximize profits [44]. However, this approach may also decrease the customer base and reduce net income. Schmalensee further expanded the two-part pricing system under the two types of consumers and non-intersecting linear demands by implementing a combined pricing strategy for the two commodities, ‘blade and razor holder’, where the manufacturer’s more durable razor holder sets a lower price [45].

The online video market in China has exhibited a trend toward monopoly, with tendencies toward an oligopolistic market structure. The number of service providers is limited, and these companies continuously innovate their business models. In pursuing profit, these enterprises urgently address the challenge of profit maximization through strategic pricing.

## Chinese internet online video pricing model

**Fig 3:**
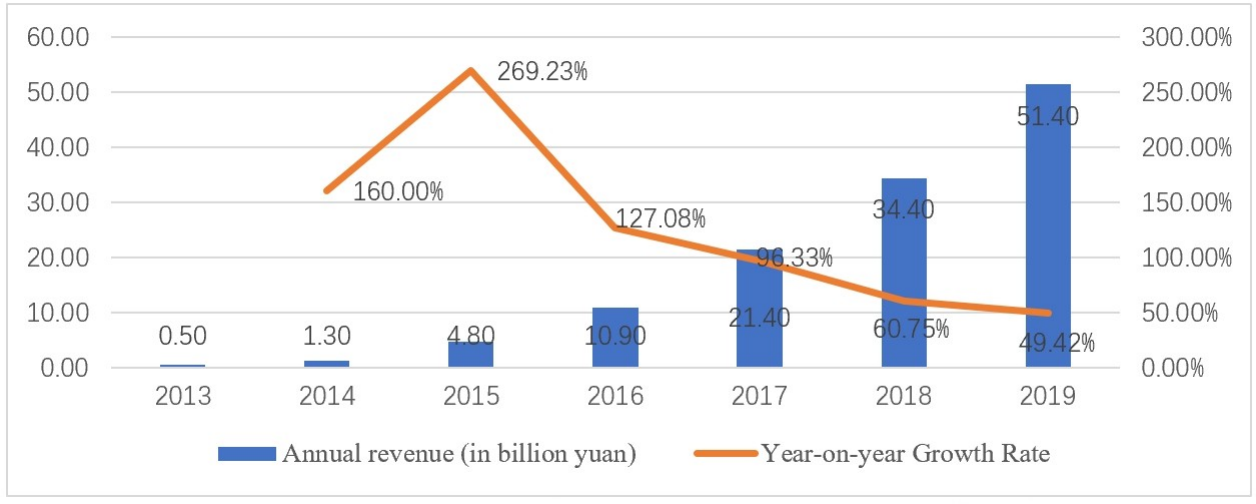
Revenue of China’s paid online video market from 2013 to 2019. Source: Analysys and collected by the author. https://www.analysys.cn/article/detail/20019731.

The paid revenue from China’s online video sector has seen swift growth due to Chinese online video companies persistently innovating new business models and operational approaches. Fig 3 illustrates the progression of revenue generated from online video payments in China, as well as the growth rate from 2013 to 2019.

A consensus has emerged from the literature regarding online video companies, highlighting that the primary sources of revenue are advertising and user payments, alongside various other profit models. Table 1 presents the breakdown of revenue streams for online video platforms in China from 2015 to 2020:

**Table 1:** Revenue distribution of the online video industry in China 2015–2020.

Year	Revenue from advertisement	Revenue from paid content	Revenue from copyright distribution	Others
2015	57.7%	12.7%	3.8%	25.8%
2016	49.0%	18.8%	3.8%	28.4%
2017	49.5%	28.1%	3.0%	19.5%
2018	47.1%	34.5%	3.0%	13.5%
2019	35.9%	42.9%	5.4%	15.8%
2020	28.6%	48.0%	5.1%	18.3%

Source: Statistic. https://www.statista.com/statistics/278574/revenue-of-chinese-online-video-industry/

The revenue from advertising primarily comes from online services in the form of brand advertising. The content payment segment includes explicit access to streaming a library of premium content, certain commercial-skipping privileges, and exclusive merchandise selection. Copyright income mainly derives from licensing content to third-party Internet video streaming platforms and collecting payments for these transactions. Other revenue streams include online gaming, live broadcasting and talent agency businesses [46]. An analysis of the income composition of various companies revealed that the revenue structure of Chinese online video companies is relatively uniform. The operating costs for these companies are primarily categorized into three groups: cost of revenues (including content and bandwidth costs), selling, general, and administrative expenses (covering promotional and marketing expenses and compensation for sales and marketing personnel), and research and development expenses (salaries and benefits for R&D personnel).

Data in Table 1 indicates a significant shift in the primary revenue sources for Chinese online video websites from 2015 to 2020. The proportion of advertising revenue decreased from 57.7% in 2015 to 28.6% in 2020, a ~30% decline. Conversely, income from user fees rose by more than 35% over five years. Online video companies feature dual-market characteristics and network externalities as part of a platform economy. Thus, pricing strategies should consider the perspectives of content providers, advertisers, and consumers. Furthermore, with user payments becoming a larger portion of revenue for online video companies, pricing strategies focusing on the consumer side are increasingly vital.

The evolution of China’s online video sector began in 2005, with most content uploaded by users. Initially, online video companies employed lenient copyright oversight to draw consumers. The China Online Video Copyright Protection Research Report in 2019 [47] from iResearch Consulting Group shows that in 2015, iQiyi revolutionized the model by offering its self-produced series "Notes from the Tomb Raiders" exclusively to paid members. It released one episode per week to free users, significantly boosting VIP membership and inaugurating the copyright payment model. Examining the membership price changes of iQiyi and Tencent Video, China’s two largest online video companies, allows for an analysis of discriminatory pricing strategies. Tables 2 and 3 detail the membership fee variations for these companies. Under the two-part pricing system, this portion of the fee is termed the entrance fee. Paid membership benefits of Chinese online video platforms typically include access to TV series, movies, and various shows, ad-free viewing privileges, Dolby Atmos, enhanced download speeds, and Blu-ray 1080P quality.

**Table 2:** iQiyi membership fee in different types.

Company’s Name	Time	Membership types	Membership Fee			(CNY)		
			Continuous Payment			Non-continuous Payment		
			Month	Quarter	Annual	Month	Quarter	Annual
**iQiyi**	Before 2020.05.23	Diamond VIP	12(49.8)	74(148)	249(498)	-	120	360
	From 2020.05.23 to 2020.11.13	Star Diamond VIP (Name changed: Diamond VIP)	40	118	398	60	-	-
	Before 2020.11.13	Golden VIP	15	45	178	19.8	58	198
	From 2020.11.13 to 2021.12.16 (The first adjusted after 9 years)	Golden VIP	19	58	218	25	68	248
		Star Diamond VIP	40	118	398	60	138	418
	From 2021.12.16 to 2022.12.15	Golden VIP	22	63	218	30	78	248
		Star Diamond VIP	40	-	398	60	-	418
	From 2022.12.16 to 2023.12.15	Golden VIP	25	68	238	30	78	258
		Star Diamond VIP	45	-	428	60	-	448

Source: From the Internet and collected by the author, prices may fluctuate due to corporate

Promotions. (https://finance.sina.cn/stock/relnews/us/2020-05-14/detail-iircuyvi3127438.d.html?from=wap, https://finance.sina.cn/tech/2020-11-13/detail-iiznezxs1617592.d.html?fromtech=1&from=wap, https://tidenews.com.cn/news.html?id=489251, https://cashier.iqiyi.com/cashier/cashier/cashier.html?vipType=4&offline=1&fv=pu_83b03f4d8e83c700&payAutoRenew=3&amount=12&pid=8287879c112d4449&skuId=sku_177079590516399139&_isolate=1)

**Table 3:** Tencent video membership fee in different types.

Company’s Name	Time	Membership types	Membership Fee			(CNY)		
			Continuous Payment			Non-continuous Payment		
			Month	Quarter	Annual	Month	Quarter	Annual
**Tencent Video**	Before 2021.04.09	Tencent Video VIP	15	45	178	20	58	198
		Super Video VIP	30	88	348	50	148	488
	From 2021.04.10 to 2022.04.19	Tencent Video VIP	20	58	218	30	68	253
		Super Video VIP	30	88	348	50	148	488
	From 2022.04.20 to 2023.12.15	Tencent Video VIP	25	68	238	30	78	258
		Super Video VIP	35	98	348	50	148	488

Source: From the Internet and collected by the author, prices may fluctuate due to corporate

Promotions. (https://www.chinatopbrands.net/s/1450-5031-26067.html, https://m.mp.oeeee.com/a/BAAFRD000020210404464077.html, https://m.mp.oeeee.com/a/BAAFRD000020220410669985.html, https://kf.qq.com/faq/220409NviINb2204092Qv2ER.html)

In November 2020, iQiyi announced its first comprehensive membership fee increase in nine years. Similarly, Tencent Video raised its membership fees in April 2021. Both companies adjusted their membership pricing in December 2021 and April 2022, respectively. According to Oi’s model, which categorizes consumers into two types, these video companies likely increased the entrance fee, *T*, to capture more consumer surplus and thus expand their revenue.

As per Oi’s profit model discussed in Section 2, the profit changes from the first type of consumers are represented in Eq (6) as −*BEF*. The changes for the second type of consumers are denoted in Eq (7) as *E*’*BFF*’. If (*BEF*) < (*E*’*BFF*’), then the company’s total profit will be greater than zero.

In their gradual adjustment of the entrance fee, online video companies have avoided abrupt price hikes to retain users with lower viewing rates. Instead, they have implemented small, frequent price increases and introduced cross-platform joint memberships to attract more paying users. The objective is to offset the loss of low-demand customers with the surplus gained from high-demand customers.

Furthermore, online video companies are continually innovating and exploring new value-added service fees, namely the pay-per-view segment. This charge primarily includes single-chip payments and advance on-demand fees.

- Single-Chip Payment Model

This model allows VIP members access to theatrical releases, major online films, European and American blockbusters, and popular, highly-rated movies on the platform. If a single-view movie incurs a copyright fee, members are offered discounts to purchase and view it or may use a limited number of movie coupons available through the VIP service. VIP members also gain early access to select episodes of high-quality copyrighted or original series and exclusive rights to self-produced paid variety shows and content not aired on popular programs. Consequently, premium VIPs enjoy more benefits compared to regular VIPs.

- Pay-Per-View in Advance Model

Companies can grow their revenue by increasing the number of paying users and maximizing consumer surplus. For instance, in June 2019, Tencent Video introduced a ’VIP Pay-per-view in Advance’ model with the successful series ’The Untamed.’ This scheme required VIP members to pay an additional 30 Yuan for early access to the final five episodes. Numerous online video platforms across China quickly adopted this payment model. Table 4 summarizes various TV series, their pricing, advanced pay-per-view rights from 2019 to 2021, and the associated video platforms.

**Table 4:** Information about some series on pay-per-view in advance from 2019 to 2021.

Broadcast Time	Drama Title	Charing Model	Broadcast Platform	Broadcast Time	Drama Title	Charing Model	Broadcast Platform
2019–06	The Untamed	Last 6 episodes, 6 Yuan per episode, 30 Yuan package	Tencent and iQiyi	2020–02	The Love Lasts Two Minds	Last 12 episodes, 3 Yuan per episode, 25 Yuan package	iQiyi
2019–12	Once Upon A Time In Lingjian Mountain	Last 5 episodes, 12 yuan package	Tencent and iQiyi	2020–03	Reborn	3 Yuan per episode, 18 Yuan package, watch 8 episodes in advance	Youku
2019–12	Joy of Life	3 yuan per episode, 50 Yuan package, always watch 6 episodes in advance	Tencent and iQiyi	2020–03	Love Of Thousand Years	3 Yuan per episode, 18 Yuan straight to the finale	Mango TV and Youku
2020–01	iPartment 5	Last 12 episodes, 3 Yuan per episode, 25 Yuan package	iQiyi	2020–03	Customer First	Last 12 episodes, 3 Yuan per episode, 25 Yuan package	iQiyi
2020–01	Great Ruler	Last 12 episodes, 3 Yuan per episode, 25 Yuan package	iQiyi	2020–03	My Roommate is a Detective	3 Yuan per episode, Last 12 episodes	iQiyi
2020–01	Ever Night 2	3 yuan/episode, 25 Yuan package, always watch 6 episodes in advance	Tencent	2020–03	Winter Begonia	3 Yuan per episode	Tencent
2020–01	Three Lives, Three Worlds, The Pillow Book	3 yuan/episode, 25 Yuan package, always watch 6 episodes in advance	Tencent	2020–03	Novoland The Castle in the Sky II	12 Yuan straight to the finale	iQiyi
2020–02	Everyone Wants to Meet You	Last 12 episodes, 3 Yuan per episode, 25 Yuan package	Tencent and iQiyi	2020–03	Imperfect Love	3 Yuan per episode, last 8 episodes	Tencent and iQiyi
2020–03	The Gutter	12 Yuan straight to the finale	Tencent and iQiyi	2020–04	Girlfriend	3 Yuan per episode	Mango TV
2020–04	The Sleuth of Ming Dynasty	Last 18 episodes, 3 Yuan per episode; 40 Yuan package	Youku	2020–04	The Best of You in My Mind	3 Yuan per episode	Youku
2020–04	If There Is No Tomorrow	Last4 episodes, 3 Yuan per episode	Youku and iQiyi	2020–04	The Love Equations	3 Yuan per episode	Tencent
2020–04	Legend of Awakening	Last 16 episodes, 3 Yuan per episode; 30 Yuan package	Mango TV and iQiyi	2020–05	Beautiful Reborn Flower	Last 24 episodes, 3 Yuan per episode	Tencent and iQiyi
2020–04	Candle in the Tomb: The Lost Caverns	3 Yuan per episode	Tencent	2020–06	The Bad Kids	3 Yuan per episode	iQiyi

Source: From the Internet and collected by the author. (https://www.yunyingpai.com/news/597631.html, https://www.toutiao.com/article/6723442347381621262/, https://finance.sina.cn/2019-12-13/detail-iihnzahi7156577.d.html, https://www.yingpingshuo.com/article/1818.html, http://news.china.com.cn/2020-07/02/content_76229919.htm, https://www.yunyingpai.com/news/597631.html)

Online video platform companies have introduced ’Pay-per-view in advance’ services to cater to users with diverse viewing habits and needs. This advanced on-demand approach aligns with the differentiated preferences of consumers, satisfying their emotional desires and allowing companies to avoid the pitfalls of homogenous competition, thereby achieving market segmentation. Additionally, by collecting data on viewers’ secondary payments, online video companies can discern the consumption preferences and tastes of various consumers, enhancing video content. Consequently, the content produced by platform companies is more closely aligned with market demands, and the targeted importation of copyrighted material fosters a continuously improving industry chain. This strategy enhances the impact of cross-network externalities in online video by offering high-quality content, which could increase the number of paying users and reduce audience disapproval of content production. As demonstrated by the previous two optimal pricing methods, the net consumer surplus, optimal marginal price, and optimal entrance fee are presented in Eqs (15), (18) and (19), respectively.

However, the implementation of ’Pay-per-view in advance’ services has met resistance from many VIP users in China and has garnered significant societal attention. On October 4, 2021, Chinese internet video giants iQiyi, Tencent Video, and Youku announced the discontinuation of the ’Pay-per-view in advance’ service and revised the additional payment terms in their VIP membership agreements. Although the service was short-lived, it is evident from the companies’ annual financial reports that it contributed to an increase in corporate revenue. iQiyi’s 2020 financial report revealed that annual revenue from membership services reached 16.5 billion yuan (approximately 2.5 billion U.S. dollars), marking a 14% increase from 2019. This growth in the membership services segment was primarily due to the implementation of diversified operational strategies, such as launching exclusive content and providing subscribers with the option to purchase on-demand content and gain early access to premium content (iQiyi, 2020). The financial benefits of the ’Pay-per-view in advance’ service under the two-part pricing system are thus evident, highlighting the viability and substantial potential for secondary payment for video content in China.

The pricing standards of internationally acclaimed online video companies, including Netflix, Disney+, HBO, Apple TV, HMVOD, and Hulu, operating in Hong Kong, China, are detailed in Table 5:

**Table 5:** Charging standard in different online video companies in 2021.

Platform	Pricing Per Month		(HKD)
Name	Basic	Standard	Premium
Netflix	$63	$78	$93
Disney+	$73		
HBO	$78		
Apple TV	$38		
HMVOD	$88		
Hulu	$55 (With Ads)	$102 (Without Ads)	

Source: From the internet and collected by the author. https://hk.trip.com/blog/streaming-platform/

In comparison, the monthly subscription fees for mainstream international online video platforms are significantly higher than those of their Chinese counterparts. Additionally, their billing items are relatively straightforward and not excessively subdivided.

## Conclusion

The robust business model of the industry is pivotal to its future development, with the profit model being an integral component. Chinese online video platforms have yet to achieve widespread profitability, making the development of pricing strategies crucial for the sustainable growth of these companies. However, the relatively nascent stage of video payment and copyright awareness in China means there is still considerable progress in refining video product pricing strategies. This paper principally examines the pricing models of iQiyi and Tencent Video, China’s leading online video enterprises, through the lens of two-part pricing systems and price discrimination theories.

Initially, drawing on the model proposed by Oi, this paper analyzes the derivation of corporate profits when charging an identical entrance and pay-per-use fee to different consumer types and the impact on profits when varying pricing strategies are employed across consumer segments. Subsequently, by deriving metrics such as consumer surplus, product demand, consumer net surplus, optimal marginal price, and optimal admission fee, this study invokes the optimal pricing formulas by scholars Tang Xiaowo and Li Keke. These formulas suggest that the entrance fee is influenced primarily by the demand function of lower-paying consumers, while optimal commodity pricing considers factors like marginal cost, consumer segmentation, and preferences.

Additionally, this paper examines the cases of iQiyi and Tencent Video, scrutinizing the logic behind their frequent membership fee adjustments and comparing these with internationally recognized online video corporations.

From the perspective of two-part pricing systems, Chinese online video firms should consider three elements to transition from losses to profits. First, companies must understand customer demand elasticity to identify the demand curves for different consumer types. By setting membership fees that cater to consumer needs, they can broaden their membership base and retain more price-sensitive customers. Second, although the single-chip payment system has been well-received by consumers, several issues regarding "advanced on-demand fees" remain unresolved, including customer charge notifications, price customization, and rights division. Moreover, these companies should proactively seek innovative business models, fostering differentiated competition and offering personalized services, which will likely trend in future development.

In recent years, the surge of short video platforms in China has altered the preferences of video consumers and commercial advertisers, impacting long video enterprises. Research on China’s long-form video companies has predominantly centered on fundamental analyses of pricing models to maximize demand and profits for content providers, platforms, and consumers. Nevertheless, with iQiyi’s membership fees increasing in December 2022 and a general convergence in fees among major long-form video companies in China, attracting consumers and monetizing traffic has emerged as a critical research direction. Additionally, a literature review indicates a dearth of scholarly work on the business model innovations of Chinese Internet long-form video enterprises, with targeted value creation studies notably absent. Future research could delve into profit sources and customer targeting. Academically, there is an opportunity to assist companies in enhancing their profit sustainability.
